# Uniportal-VATS for Early-Stage NSCLC in Octogenarians: A Single-Center, Retrospective Study of Surgical and Short-Term Oncological Outcomes

**DOI:** 10.3390/jpm16030155

**Published:** 2026-03-07

**Authors:** Dania Nachira, Alessia Senatore, Giovanni Punzo, Maria Letizia Vita, Maria Teresa Congedo, Khrystyna Kuzmych, Leonardo Petracca-Ciavarella, Filippo Lococo, Elisa Meacci, Stefano Margaritora

**Affiliations:** 1 Department of General Thoracic Surgery, Fondazione Policlinico Universitario “A. Gemelli”, IRCCS, Università Cattolica del Sacro Cuore, 00168 Rome, Italy; danynac@libero.it (D.N.); alessia.senatore01@icatt.it (A.S.); mariateresa.congedo@policlinicogemelli.it (M.T.C.); leonardo.petraccaciavarella@policlinicogemelli.it (L.P.-C.); filippo.lococo@policlinicogemelli.it (F.L.); elisa.meacci@unicatt.it (E.M.); stefano.margaritora@policlinicogemelli.it (S.M.); 2 Department of Anesthesiology and Intensive Care Medicine, Fondazione Policlinico Universitario “A. Gemelli”, IRCCS, Università Cattolica del Sacro Cuore, 00168 Rome, Italy; giovanni.punzo@policlinicogemelli.it

**Keywords:** octogenarians, elderly, NSCLC, Uniportal-VATS, outcomes

## Abstract

**Background/Objectives:** This study aimed to assess the safety and efficacy of lung surgery for the treatment of early-stage non-small cell lung cancer (NSCLC) in octogenarians, with a specific focus on the Uniportal-VATS approach, evaluating surgical outcomes and short-term oncological results within a precision medicine perspective. **Methods**: This retrospective, single-center study included octogenarian patients who underwent surgical treatment for early-stage NSCLC between January 2018 and March 2024. Among 1329 patients treated during the study period, 136 octogenarians were carefully evaluated by a multidisciplinary board and selected for surgical management. **Results**: The mean age was 82.41 ± 2.72 years, with a prevalence of men (63.2%). In 107 (78.7%) cases, lung resection was performed using the Uniportal-video-assisted thoracic surgery (U-VATS) approach. Overall, 71 lobectomies (52.2%) and 65 segmentectomies or wedge resections (47.8%) were performed, balancing oncological radicality with comorbidities. Only minor complications occurred, such as atelectasis (2.9%), atrial fibrillation (4.4%), pneumonia (1.5%), or air-leakage (2.2%). Factors significantly associated with postoperative complications included open approach (*p* = 0.014), lobectomy as the extent of resection (*p* = 0.008), and chronic obstructive pulmonary disease (COPD) (*p* = 0.010). On multivariable analysis, lobectomy remained the only independent predictor for postoperative complications (OR: 5.95, 95% CI [1.24–28.62], *p* = 0.026). In-hospital and 90-day mortality were null. The median length of hospital stay in octogenarians was 6 days and was significantly shorter in the Uniportal-VATS group compared with the open surgery one (*p* < 0.001). All patients were discharged home independently. One- and three-year overall survival rates were 88% and 71%, respectively. No risk factor was associated with mortality in our series. **Conclusions**: Lung surgery, particularly the Uniportal-VATS approach, appears to be a safe and effective treatment option for octogenarian patients with early-stage NSCLC, provided that patient selection is carefully based on individual clinical characteristics within a multidisciplinary framework based on individualized risk stratification. When feasible, sublobar resection should be preferred in order to minimize postoperative complications.

## 1. Introduction

The increase in life expectancy has led to a rising incidence of lung cancer, even among elderly patients, resulting in a growing number of non-small cell lung cancer (NSCLC) diagnoses in subjects aged ≥80 years [1]. Although surgical resection is the first-line treatment for early-stage NSCLC, advanced age and the presence of comorbidities, particularly cardiovascular and respiratory ones, make surgery very challenging in this subset of patients, which is frequently classified as being at high perioperative risk [2]. Indeed, octogenarians often exhibit reduced cardiopulmonary function, lower postoperative pain tolerance, and slower postoperative recovery, which together increase the risk of postoperative cardiovascular and respiratory complications compared with younger patients. Consequently, the risk associated with surgery is usually much higher in elderly patients with NSCLC [3].

In recent years, minimally invasive thoracic approaches, such as video-assisted thoracoscopic surgery (VATS), have been associated with superior perioperative outcomes when compared to open thoracotomy, with numerous articles and meta-analyses demonstrating reduced complication rates, shorter hospital stay, and improved long-term survival [4,5,6].

In this context, Uniportal-VATS has emerged as an even less invasive alternative to the multiportal approach, with promising results in terms of reduced surgical trauma, better postoperative pain control, limited impact on respiratory function, and shorter hospital stays compared with conventional thoracotomy and multiportal-VATS [7,8,9].

Nonetheless, evidence regarding the safety and oncological efficacy of this technique in octogenarian patients with early-stage NSCLC remains limited and inconclusive, particularly concerning oncological outcomes.

The present study, therefore, aims to assess the safety and efficacy of lung surgery in carefully selected octogenarian patients with early-stage NSCLC, with a specific focus on the Uniportal-VATS approach, by evaluating surgical outcomes and short-term oncological results.

## 2. Materials and Methods

### 2.1. Study Design

This is a retrospective, single-center cohort study conducted between January 2018 and March 2024 on octogenarian patients with early-stage (cT1a-T2bN0M0) NSCLC who underwent surgical treatment at our center. The study was approved by the Institutional Review Board of Università Cattolica del Sacro Cuore (Approval Code: ID6387; Approval Date: April 2024) and conducted in accordance with the principles of the Declaration of Helsinki. All patients provided informed consent for surgical treatment and the anonymous use of clinical data for research purposes. Data collection and reporting adhered to the Strengthening the Reporting of Observational Studies in Epidemiology (STROBE) guidelines.

Among 1329 patients who underwent surgical resection for early-stage NSCLC at our center during the study period, 136 octogenarians (≥80 years old) were identified, representing 10.2% of the total cohort.

The inclusion criteria were:

- Age ≥ 80 years;
- Histologically confirmed diagnosis of early-stage NSCLC;
- Surgical resection performed with curative intent;
- Availability of complete clinical and follow-up data.

The exclusion criteria included:

- Advanced or metastatic disease;
- Patients treated with non-surgical therapies as a first-line treatment.

### 2.2. Preoperative Assessment

All patients were carefully evaluated by a multidisciplinary team including thoracic surgeons, anesthesiologists, pulmonologists, and oncologists. The decision to proceed with surgery was based on a comprehensive and individualized preoperative assessment and meticulous patient selection, including evaluation of cardiological and functional status, comorbidities, anesthetic risk (ASA score), and respiratory function (complete pulmonary function testing) to predict surgical tolerance. For staging, all patients underwent a contrast-enhanced total-body computed tomography (CT), with whole-body positron emission tomography (PET) performed when clinically indicated. Whenever feasible, a preoperative histological diagnosis was obtained by bronchoscopic fine-needle aspiration (FNA), often guided by endobronchial ultrasound, or by CT-guided FNA, according to the specific case.

### 2.3. Surgical Techniques

A Uniportal-VATS approach, which represented the standard minimally invasive technique at our center during the study period, was adopted whenever feasible. Thoracotomy was planned in selected patients presenting with more complex anatomical or clinical characteristics (e.g., large or perihilar masses), particularly during the first two years of the study period, at the beginning of our Uniportal-VATS experience. Uniportal-VATS procedures were performed through a single 3–4 cm incision, most commonly located in the fifth intercostal space. When necessary, the incision was shifted to the fourth or sixth intercostal space according to lesion characteristics and patient anthropometry. A wound retractor was routinely applied, and a 30° thoracoscope was positioned in the superior portion of the incision. Dedicated long and curved thoracoscopic instruments with proximal and distal articulation, together with endoscopic stapling devices, were introduced through the same access. The curved design of the instruments allowed the simultaneous introduction of multiple tools through the small incision, while minimizing instrument fencing [10,11].

The choice of surgical resection was determined by multidisciplinary criteria, balancing oncological radicality with functional reserve and comorbidities. Sublobar resection, such as segmentectomy or wedge resection, was generally indicated for small (<2 cm), peripheral, or subsolid pulmonary nodules, particularly in the presence of significant comorbidities or limited functional reserve. In the presence of these conditions precluding lobectomy, technically demanding complex segmentectomies, particularly those involving the lower lobes, were generally avoided in octogenarians. In such cases, wedge resection was preferred in order to shorten operative time and reduce the risk of complications associated with prolonged anesthesia.

Systematic hilar and mediastinal lymph node dissection was routinely carried out in all resections, regardless of the surgical approach.

In the Uniportal-VATS approach, a single 28-Fr chest drain was placed at the end of the procedure, whereas two chest drains were typically used in the open approach. Chest tube removal was generally performed on postoperative day 3, provided that the drainage was less than 250–300 mL over the preceding 24 h, no air leak was detected, and postoperative chest radiography confirmed complete lung re-expansion.

### 2.4. Postoperative Management

Postoperative analgesia was provided using locoregional techniques, including paravertebral blocks, intercostal nerve blocks, and fascial plane (erector spinae plane and serratus anterior plane) blocks. No patient received thoracic epidural analgesia [12,13].

After surgery, patients were transferred to a monitored recovery unit for close surveillance of pain control, respiratory function, and hemodynamic status. Enhanced Recovery After Surgery (ERAS) pathways were systematically implemented to facilitate postoperative recovery, including early mobilization, physiotherapy, and respiratory exercises.

### 2.5. Outcomes

The primary outcomes of the study were to evaluate intra- and postoperative complication rates, as well as in-hospital and 90-day mortality, in the overall cohort of octogenarian patients and specifically in the Uniportal-VATS versus open surgery groups.

The secondary outcomes included length of hospital stay, identification of predictors of complications and mortality, and short-term overall survival at 1 and 3 years in the entire study population.

### 2.6. Statistical Analysis

Continuous data are reported as mean ± standard deviation (SD) for normally distributed variables, or as median with interquartile range (IQR) for non-normally distributed variables. Data normality was evaluated using the Shapiro–Wilk test. Comparisons between continuous variables were conducted using the independent-samples Student’s *t* test, with correction for unequal variances when appropriate, or the Mann–Whitney U test in the absence of normal distribution.

Categorical variables are expressed as counts and percentages and were compared using the chi-square test.

Univariable and multivariable regression analyses were performed to identify risk factors associated with complications. Variables with a *p*-value < 0.20 in univariable analysis were included in the multivariable model. Survival probabilities were estimated using the Kaplan–Meier method and compared by means of the log-rank test. A multivariable Cox proportional hazards model was used to assess risk factors associated with mortality.

All statistical tests were two-sided, and a *p*-value < 0.05 was considered statistically significant. Statistical analyses were carried out using IBM SPSS Statistics for Macintosh, version 25.0 (IBM Corp., Armonk, NY, USA).

## 3. Results

A total of 136 octogenarian patients were selected among a series of 1329 patients who underwent surgery for NSCLC at our center during the study period. The mean age of octogenarians was 82.41 ± 2.72 years (median = 82). Eighty-six (63.2%) were male. The main clinical characteristics of the study population are reported in Table 1.

Overall, the Uniportal-VATS approach was used in 78.67% of cases (107 patients), as shown in Table 1.

Seventy-one patients (52.2%) underwent pulmonary lobectomy, which was performed using the Uniportal-VATS approach in 81.4% of cases. In the remaining 65 patients (47.8%), a sublobar resection was performed (a wedge resection in 39 cases (28.7%), while a segmentectomy in 26 (19.1%)).

### 3.1. Surgical Outcomes

The overall complication rate in octogenarians was 17.6% (24 cases). In particular, all complications occurred in the postoperative period and were classified as minor (grade I–II) according to Clavien–Dindo Classification (see Table 2). No intraoperative adverse events were recorded.

Postoperative complications were more frequent in the open surgery group than in the Uniportal-VATS group (34.5%vs.13.1%, *p* = 0.014), mainly due to a higher incidence of pulmonary complications, including atelectasis (*p* = 0.010) and pneumonia (*p* = 0.007), Table 2. Overall, the mean length of hospital stay was 5.53 ± 5.33 days (median = 4) and was significantly shorter in the Uniportal-VATS group (*p* < 0.001), Table 2.

All elderly patients were discharged home independently, with no need for rehabilitation facilities. Both in-hospital and 90-day mortality were null.

When evaluating the main variables associated with a higher risk of complications in the entire series of octogenarians, only an open approach (*p* = 0.014), lobectomy as the extent of resection (*p* = 0.008), and COPD (*p* = 0.010) showed statistical significance. However, on multivariable analysis, lobectomy remained the only factor significantly associated with postoperative complications (OR: 5.95, 95%CI [1.24–28.62], *p* = 0.026), Table 3.

### 3.2. Short-Term Oncological Outcomes

One- and three-year overall survival (OS) rates in octogenarians were 88% and 71%, respectively. Interestingly, no difference was found when these outcomes were compared with those of our historical cohort of younger patients operated on during the same study period (1-year and 3-year OS: 91% and 75%, *p* = 0.182). On univariable analysis, no clinical or surgical factors were associated with overall mortality in elderly patients.

## 4. Discussion

The results of the present study confirm lung surgery as a valuable option for carefully selected octogenarian patients with early-stage NSCLC. In particular, minimally invasive surgery (MIS), such as Uniportal-VATS, could represent a safe and effective surgical strategy for reducing surgical complications and enhancing postoperative recovery in elderly patients. Despite the high fragility profile of this population, often characterized by respiratory and cardiovascular comorbidities, the overall incidence of postoperative complications remained lower in the Uniportal-VATS group, and no 90-day mortality was observed in the entire cohort.

The routine adoption of the Uniportal-VATS technique, used in 81.4% of cases, substantially contributed to reduced surgical trauma, promoting earlier functional recovery and facilitating earlier discharge home for all patients. These results are in line with growing evidence supporting the use of minimally invasive surgery in elderly patients, which has been associated with improved tolerability compared to the traditional approach.

In support of these data, a French study analyzing an administrative database reported a crude 30-day mortality rate of 3.85% among octogenarians operated with the VATS approach, compared with 7.9% (*p* < 0.0001) in those treated with the traditional thoracotomy approach [14].

Similarly, Bravo Iniguez et al. compared postoperative outcomes following lobectomy for NSCLC in elderly patients (>65 years) by analyzing the ACS-NSQIP database. A significantly lower 30-day mortality was found after VATS lobectomy (1.19%) compared with open lobectomy (3.13%), without a significant increase in mortality among patients aged 75–80 years [15].

More recently, Detillon et al., analyzing data from the Dutch Lung Surgery Database, where approximately 85% of patients underwent lobectomy, reported a significantly higher postoperative mortality in octogenarians (6%) compared with patients aged 60–69 (1%) and 70–79 years (2.6%). Notably, they also reported reduced odds of mortality for patients treated with the minimally invasive VATS approach [16].

Furthermore, Bongiolatti and colleagues [17] highlighted a significantly higher incidence of postoperative complications in octogenarian patients, along with longer chest tube duration and length of hospital stay. In particular, octogenarians were more prone to develop complications such as atrial fibrillation, pneumonia, hemothorax, and the need for blood transfusions. Although octogenarians experienced a higher rate of complications, these events were predominantly not life-threatening, as demonstrated by the comparable 90-day mortality with the younger population (1.3% in octogenarians vs. 1.2% in younger patients). These findings support the safety of VATS in carefully selected octogenarian patients [17].

Overall, the available literature supports the safety and effectiveness of MIS for the treatment of NSCLC in octogenarians. Within this context, increasing attention has been directed toward robotic-assisted thoracic surgery (RATS). Pan H. et al. compared RATS and VATS approaches for sublobar resection in octogenarians with stage IA NSCLC and reported that RATS was associated with lower intraoperative blood loss and a shorter postoperative hospital stay (4 days vs. 5 days). However, the two groups were comparable in terms of surgical duration, conversion rate, blood transfusion, chest tube volume, and duration, as well as other perioperative outcomes and postoperative complications. Additionally, after a median follow-up of 66 months, RATS and VATS achieved comparable outcomes, including 5-year overall survival, recurrence-free survival, and cumulative incidence of death [18]. 

In our series, multivariable analysis showed that lobectomy was significantly associated with an increased risk of postoperative complications, emerging as the only independent risk factor in the octogenarian population (*p* = 0.026). This finding reinforces the importance of individualized surgical planning, in which the balance between oncological radicality and minimization of clinical risk becomes central. The high percentage of sublobar resections (segmentectomies and atypical resections, 47.8%) reflects this personalized strategy, particularly indicated in patients with reduced functional reserve or multiple comorbidities [19]. In the elderly population, the potential benefit of radical surgical treatment may decrease with age due to reduced life expectancy and diminished tolerance of stress [20].

When selecting the optimal surgical strategy for these patients, frailty must be carefully considered, with the aim of achieving the best possible outcome while minimizing physiological stress. Given that the primary goal of segmentectomy is the preservation of pulmonary function [21], this approach is appropriate, whenever feasible, in octogenarian patients with impaired respiratory function and early-stage disease. In selected cases, wedge resection may also be considered when feasible, particularly when, based on our experience, a complex segmentectomy could offset the potential benefits of a sublobar resection by increasing the risk of complications associated with prolonged anesthesia or technically demanding procedures. Such strategies allow oncological radicality to be maintained while reducing surgical risk and promoting a faster and more favorable postoperative recovery [22], particularly when associated with a minimally invasive approach.

Although octogenarian patients generally experienced a longer average hospital stay, the recovery of preoperative functional autonomy and the observed 1- and 3-year survival rates (88% and 71%) indicate that chronological age, in the absence of severe biological frailty, does not constitute itself an absolute contraindication to surgery for early-stage NSCLC in well-selected patients [23]. Moreover, MIS was associated with significantly shorter postoperative hospitalization (*p* < 0.001).

Interestingly, Singh et al. [24] showed how lung cancer in octogenarian patients is frequently diagnosed at an advanced stage and that, even among patients with early-stage disease, surgery is less often offered as first-line treatment, due to advanced age, comorbidities, and concerns regarding potential adverse outcomes.

These observations highlight the importance of careful patient selection in mitigating the potential surgical risks in octogenarian candidates. Thorough preoperative evaluation, accurate prediction of their postoperative functional status, and optimized postoperative care are essential components of the decision-making process.

Furthermore, in this setting, in recent years, the adoption of the ERAS protocol and MIS has played a crucial role. In our experience at a high-volume center, this strategy has guided the surgical management of octogenarians with early-stage NSCLC. Specifically, meticulous patient selection, the adoption of MIS [25], particularly the less invasive Uniportal-VATS approach, and the implementation of advanced analgesic protocols based on regional nerve/fascial blocks [12,13] have contributed to reduced surgical trauma and postoperative pain, thereby enhancing patient recovery and allowing a greater number of elderly patients to be treated with satisfactory outcomes.

The ERAS concept relies on close collaboration among multiple disciplines, such as anesthesia, nursing, and surgery, and encompasses measures such as preoperative education and preparation, maintenance of normothermia, use of anesthetics with a shorter half-life, restrictive intraoperative fluid replacement, and early postoperative mobilization. In this regard, a study by Huang et al. [26] showed that adopting ERAS during the perioperative period of Uniportal-VATS can reduce postoperative pain, shorten postoperative hospital stay, and decrease chest tube duration.

In this context, our results highlight the importance of an individualized, risk-adapted surgical strategy in octogenarians, moving beyond chronological age toward a more personalized decision-making model.

Building on these considerations, the integration of artificial intelligence (AI) into clinical practice may further refine the decision-making process for octogenarian candidates for lung resection. By leveraging large-scale clinical, radiological, and functional datasets, AI-based predictive models could improve risk stratification, provide more accurate estimates of postoperative functional decline, and support individualized assessments of surgical tolerance. Machine learning algorithms may assist in identifying subtle patterns of frailty, comorbidity burden, and physiological reserve that are not fully captured by conventional risk scores, thereby helping to determine not only whether an elderly patient should undergo surgery, but also which surgical strategy, lobectomy, segmentectomy, or wedge resection, would offer the most favorable balance between oncologic efficacy and perioperative safety. Furthermore, AI-driven tools could optimize perioperative planning within ERAS pathways, tailoring anesthetic management, analgesic strategies, and postoperative care to the specific risk profile of each patient. In this evolving scenario, AI should be regarded as a complementary instrument to multidisciplinary clinical judgment, with the potential to enhance precision, standardize decision-making, and ultimately expand safe surgical indications in carefully selected elderly patients with early-stage NSCLC.

Therefore, in a population with increasing life expectancy, our preliminary results suggest that surgical treatment may be considered in elderly patients when surgery represents a potentially curative option. Nevertheless, careful evaluation of elderly patients and a structured care pathway including ERAS, MIS protocols, and, in the near future, AI-based predictive models must be mandatory to reduce postoperative complications and minimize the risk of loss of independence [27], which represents a key modifiable factor in such frail patients.

### Limitations and Point of Strengths

This study has some limitations, including its single-center design, the relatively limited sample size, and the highly selected patient population, which may limit the generalizability of the results. In particular, the small number of elderly patients treated via thoracotomy may limit the findings of the subgroup analyses. Moreover, a longer oncological follow-up is required to fully evaluate the long-term oncological efficacy of minimally invasive surgery in this population.

Despite these limitations, the study also has strengths. These include one of the largest reported single-center series of octogenarian patients treated predominantly with a Uniportal-VATS approach, the consistent application of a multidisciplinary management strategy, and the systematic implementation of ERAS and advanced analgesic protocols in a particularly frail patient population.

## 5. Conclusions

In conclusion, lung surgery, particularly the Uniportal-VATS approach when associated with ERAS protocols and multidisciplinary patient selection, appears to be a valid and safe therapeutic option for octogenarian patients with early-stage NSCLC. When feasible, sublobar resection should be preferred in order to minimize postoperative complications. These findings support a personalized, risk-adapted surgical strategy in elderly patients. Further prospective studies are warranted to validate these results and refine patient selection criteria.

## Figures and Tables

**Table 1:** Clinical characteristics of the study population of octogenarian patients undergoing surgery for early-stage NSCLC.

	Octogenarian Patients (n = 136)
Mean age (years)	82.41 ± 2.72
Male sex	86 (63.2%)
Current or former smokers	97 (71.3%)
COPD	68 (50.0%)
Cardiovascular comorbidities	65 (47.7%)
Diabetes	35 (25.7%)
Histology (adenocarcinoma)	100 (73.5%)
Tumor dimension (cm)	2.77 ± 1.56
Lung resection: - Lobectomy - Segmentectomy/wedge resection	71 (52.2%) 65 (47.8%)
Surgical approach: - Thoracotomy - Uniportal-VATS	29 (21.3%) 107 (78.7%)
Preoperative PO_2_ (mmHg)	83.59 ± 11.66
Preoperative CO_2_ (mmHg)	39.93 ± 6.60
Preoperative FEV1%	96.06 ± 21.97
Preoperative FVC%	101.98 ± 22.36

Abbreviations: COPD, chronic obstructive pulmonary disease; FEV1, forced expiratory volume in 1 s; FVC, forced vital capacity; PO_2_, partial pressure of oxygen; CO_2_, partial pressure of carbon dioxide.

**Table 2:** Postoperative outcomes in Uniportal-VATS versus open surgery groups (in bold, significant values with *p* < 0.05).

	Patients (n = 136)	Uniportal-VATS (n = 107)	Open (n = 29)	*p*
**Postoperative complications:**	24 (17.6%)	14 (13.1%)	10 (34.5%)	**0.014**
- Atelectasis	4 (2.9%)	1 (0.9%)	3 (10.3%)	**0.010**
- Atrial fibrillation	6 (4.4%)	6 (5.6%)	3 (10.3%)	0.410
- Pneumonia	2 (1.5%)	0 (0%)	2 (6.9%)	**0.007**
- Prolonged air-leak (>5 days)	3 (2.2%)	3 (2.8%)	0 (0%)	0.487
- Bleeding	2 (1.5%)	1 (0.9%)	1 (3.4%)	0.343
**Length of hospital stay,** mean ± SD (median), days	5.53 ± 5.33 (4)	4.64 ± 2.04 (4)	8.64 ± 10.41 (6)	**<0.001**
**30-day mortality**	0 (0%)	0 (0%)	0 (0%)	1.00
**90-day mortality**	0 (0%)	0 (0%)	0 (0%)	1.00

Abbreviations: SD, standard deviation; VATS, video-assisted thoracoscopic surgery.

**Table 3:** Univariable and multivariable analyses of risk factors associated with postoperative complications (in bold, significant values, *p* < 0.05).

	Univariable Analysis	Multivariable Analysis	
	*p*-Value	OR [95% CI]	*p*-Value
Male sex	0.197		
Smoking	0.318		
COPD	**0.010**		
Diabetes mellitus II	0.460		
Cardiovascular diseases	0.775		
FEV1%	**0.010**		
FVC%	**0.019**		
Lobectomy	**0.008**	5.95 [1.24–28.62]	**0.026**
Open approach	**0.014**		

Abbreviations: OR, odds ratio; CI, confidence interval; FEV1, forced expiratory volume in 1 s; FVC, forced vital capacity; COPD, chronic obstructive pulmonary disease.

## Data Availability

The datasets generated and/or analyzed during the current study are available from the corresponding authors on reasonable request.

## Abbreviations

The following abbreviations are used in this manuscript:

NSCLC	Non-Small Cell Lung Cancer
VATS	Video-Assisted Thoracoscopy Surgery
M-VATS	Multiportal-Video-Assisted Thoracoscopy Surgery
U-VATS	Uniportal-Video-Assisted Thoracoscopy Surgery
ERAS	Enhanced Recovery After Surgery
c-SAPB	continuous superficial serratus anterior plane block
ICNB	intercostal nerve block
c-ESPB	erector spinae plane block
RATS	Robot-assisted thoracoscopy
MIS	minimally invasive surgery
